# Effect of tylosin on dogs with suspected tylosin-responsive diarrhea: a placebo-controlled, randomized, double-blinded, prospective clinical trial

**DOI:** 10.1186/1751-0147-53-26

**Published:** 2011-04-14

**Authors:** Susanne Kilpinen, Thomas Spillmann, Pernilla Syrjä, Teresa Skrzypczak, Maria Louhelainen, Elias Westermarck

**Affiliations:** 1 Department of Equine and Small Animal Medicine, Faculty of Veterinary Medicine, University of Helsinki, Helsinki, Finland; 2 Department of Veterinary Biosciences, Faculty of Veterinary Medicine, University of Helsinki, Helsinki, Finland; 3 Research Department, Veterinary Bacteriology, Finnish Food Safety Authority, Helsinki, Finland

## Abstract

**Background:**

The macrolid antibiotic tylosin has been widely used to treat canine chronic diarrhea, although its efficacy is based on anecdotal reports and experimental studies in dogs and not on strong scientific evidence. The term tylosin-responsive diarrhea (TRD) refers to diarrheal disorders responding to tylosin therapy within a few days. In TRD, the stool remains normal as long as tylosin treatment continues, but diarrhea reappears in many dogs within weeks after discontinuation. The aim of our trial was to assess the effect of tylosin on fecal consistency compared with a placebo treatment in dogs with suspected TRD and additionally to establish whether tylosin in dogs with recurrent diarrhea is as effective as empirical studies and anecdotal reports suggest.

**Methods:**

Subjects comprised 71 client-owned dogs that, according to the owners, had previously been treated successfully with tylosin due to recurrent diarrhea of unknown etiology. At the initial examination, where there were no signs of diarrhea, the dogs were randomly assigned in a 2:1 ratio to a tylosin or placebo group. During a two-month follow-up the owners evaluated the fecal consistency according to previously published guidelines. When diarrhea recurred, either tylosin (25 mg/kg q 24 h, 7 days) or placebo treatment was initiated orally. Treatment outcome was evaluated as the mean of fecal consistency scores assigned during the last three days of the treatment period. To test for differences between the tylosin and placebo group in the proportion of responders, Pearson's Chi-squared test and Fisher's exact test were applied.

**Results:**

Sixty-one dogs met the selection criteria and were followed for two months. During the follow-up 27 dogs developed diarrhea and either tylosin or placebo treatment was started. The proportion of dogs with normal fecal consistency at the end of treatment was 85% (17/20) in the tylosin group and 29% (2/7) in the placebo group (Pearson's Chi-squared test p = 0.0049 and Fisher's exact test two-sided, p = 0.0114).

**Conclusions:**

Our findings indicate that tylosin is effective in treating recurrent diarrhea in dogs. The dose of 25 mg/kg once daily appears sufficient. No changes specific to TRD were detected in the examinations.

## Background

The macrolid antibiotic tylosin, launched on the drug market in Finland already 40 years ago in tablet form under the trade name Tylan ^®^, was marketed as a useful medicine for the treatment of different bacterial infections in dogs. Finnish veterinarians noted that tylosin was an effective drug in the treatment of canine diarrhea. About ten years ago, the tablet form of the drug was recalled, as the manufacturer was interested mainly in marketing tylosin in powder form for pigs and poultry. Since then, the University Pharmacy in Finland has dispensed tylosin in tablet form on request, commonly to treat canine diarrheal disorders. However, usage has been based on anecdotal reports of owners, rather than on strong scientific evidence. Recently, the effect of tylosin was shown to differ from that of other antibiotics, and the term tylosin-responsive diarrhea (TRD) was proposed for diarrheal disorders responding to tylosin therapy within a few days [1,2]. In TRD dogs, stool is normal as long as treatment continues, but diarrhea reappears in many dogs within weeks after discontinuation [1]. The owners of the dogs reported that even after several treatments the effect of tylosin was as good as at the initial treatment. This is astonishing, as tylosin is an antibiotic substance, and long-term usage of antibiotics generally contributes to the development of microbial resistance. Usually, achieving a positive antibiotic treatment is even more difficult if the same antibiotic is used repeatedly to treat the same disease in the same individual.

However, these noncontrolled studies [1,2] were conducted with only a small number of dogs, and whether tylosin is really as effective as suggested is uncertain. The diarrhea might resolve by itself, as we know that in chronic diarrhea patients the symptoms are often fluctuating, and these patients may become temporarily asymptomatic without any treatment [3]. To close the gap between empirical studies and evidence-based medicine, we performed a placebo-controlled, randomized, double-blinded, prospective clinical trial. The aims were to assess the effect of tylosin on fecal consistency compared with a placebo treatment in dogs with suspected TRD and to establish whether tylosin treatment in canine patients with recurrent diarrhea is as effective as noncontrolled studies and anecdotal reports suggest.

## Methods

**Figure 1:**
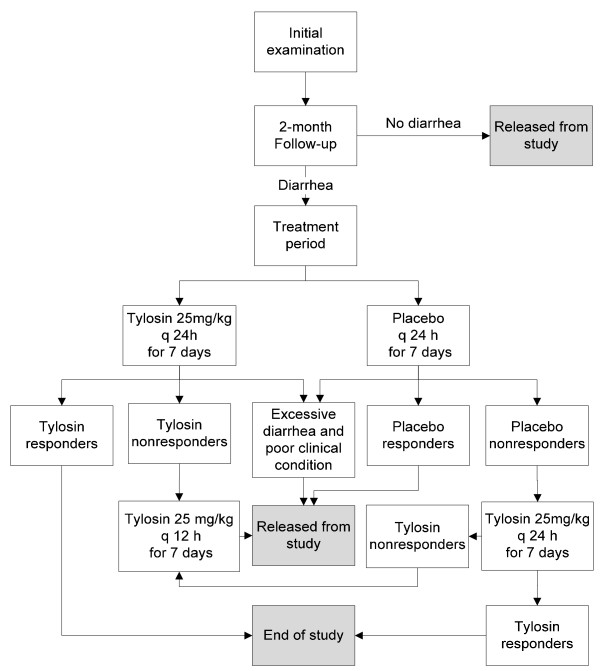
**Study protocol including enrollment, allocation to treatment groups, and treatment of suspected TRD dogs**.

Over the period of October 2006 and April 2008, 71 client-owned dogs with recurrent diarrhea were referred to the trial at the Small Animal Hospital, Faculty of Veterinary Medicine, University of Helsinki, Finland. The owners signed a written informed consent in which they agreed to let their dog participate and gave permission for collection of blood and fecal samples and performance of a gastroduodenoscopy on their dogs. The study protocol (Figure 1) was approved by both the National Animal Ethics Committee in Finland and the Ethics Committee for Animal Experiments of the University of Helsinki, Finland.

### Selection criteria

One selection criterion was a previous successful empiric tylosin treatment of the dog for recurrent diarrhea of unknown etiology. Suspected TRD dogs were over six months old and had not been treated with systemic corticosteroids, nonsteroidal anti-inflammatory drugs, or antibiotics other than tylosin in the 30 days preceding the trial. At the initial examination, the dogs were either on current tylosin medication or the tylosin treatment had been discontinued within the last four weeks. Pregnant and lactating females were excluded, as were dogs with evidence of clinically important systemic or organ-related disease that secondarily could cause diarrhea. The dogs had no clinical signs of diarrhea at the initial examination.

### Initial examination

At the initial examination, the dogs were randomly allocated to two treatment groups. A block randomization with a block size of 15 was utilized. According to a skewed randomization, within each block two-thirds of dogs were assigned to the tylosin group and one-third to the placebo group, receiving, respectively, either tylosin tartrate tablets at a dose of 25 mg/kg or microcrystalline cellulose tablets orally once a day for seven days. To ensure fulfillment of the selection criteria, the initial examination was performed as follows. A clinical history and physical examination were carried out. Blood samples were collected for determination of complete blood count (CBC), serum alanine transaminase and alkaline phosphatase activities, and urea, creatinine, glucose, total protein, albumin, sodium, potassium, cobalamin, folate, and trypsin-like immunoreactivity concentrations. Urinalysis for specific gravity, dipstick, and sediment was performed. Fecal samples from three consecutive days were analyzed for endoparasite ova using the magnesium-sulfate flotation method. A fresh fecal sample collected manually from the rectum was stored at -20°C and examined by a solid-phase immunoassay (ProSpecT *Giardia *Microplate Assay, Remel Europe Ltd., Dartford, United Kingdom) for the detection of *Giardia *spp. The Central Laboratory of the Department of Equine and Small Animal Medicine carried out all the analyses for routine CBC and serum parameters, urinalysis and the parasitological analyses of the fecal samples. The laboratory is equipped with modern clinical chemistry and hematology analyzers. Serum samples of serum TLI, folate, and cobalamin concentrations were sent for analysis to a commercial laboratory (Vetlab Oy, Tampere, Finland).

Dogs with cobalamin concentrations under the reference range were treated with subcutaneous injections of cobalamin after release from the trial.

Gastroduodenoscopy was performed and six mucosal biopsy specimens were obtained from the duodenum. A histopathological examination of the biopsies was performed by a single European College of Veterinary Pathology board-certified pathologist. The biopsies were examined for severity and type of inflammation according to the World Small Animal Veterinary Association (WSAVA) Gastrointestinal Standardization Group`s international standards [4,5]. Severity of inflammation was graded as none, mild, moderate, or severe, and type of inflammation as none, lymphoplasmacytic, eosinophilic, or other.

### Follow-up period

Directly after the initial examination, all dogs were treated with fenbendazole 50 mg/kg once a day for three days.

Ten days after the initial examination, a follow-up period of a maximum of two months commenced to determine whether diarrhea would appear. Tylosin medication was discontinued in the dogs receiving it at the time of the initial examination. Throughout the study, the feeding management and diet of all dogs remained unchanged from those at the initial examination.

The owners evaluated and recorded the fecal consistency of each stool specimen according to previously published guidelines using a nine-point scale, from one to five, with half-point intervals [1,2,6]. Instructions on how to evaluate consistency were provided to owners in both picture and written form to facilitate and standardize the subjective evaluation as far as possible. The photos were explicit, enabling owners to evaluate the fecal consistency adequately. Scores 5 and 4.5 were considered unacceptable. Score 4 feces were of poor quality; they were very moist and poorly formed, with the consistency of putty or porridge. Moist feces, which nevertheless had some definite form, were scored as 3.5. Score 3 represented feces of good quality that were slightly moist. Ideal feces were those that could easily be picked up and left no stain (scores 2.5 and 2). In addition, the owners evaluated daily the severity of the following clinical signs: alertness (normal or reduced), appetite (normal, reduced, or considerably reduced), borborygmus (no, occasionally, or often), flatulence (no, occasionally, or often), and vomiting (no, occasionally, or often). If diarrhea did not reappear during the two-month follow-up, the dogs were released from the study (Figure 1).

### Treatment period

Dogs with reappearance of diarrhea (fecal consistency score ≥4) went through the same procedures as in the initial examination, excluding the gastroduodenoscopy. In addition, a fresh fecal sample was collected manually from the rectum and cultured at the Veterinary Bacteriology, Research Department of the Finnish Food Safety Authority, Helsinki, Finland, for *Yersinia *spp., *Salmonella *spp., *Campylobacter *spp., *Clostridium difficile, and Clostridium perfringens*. Either tylosin or placebo treatment was then initiated. Both the investigator and owner were blinded to treatment. Information about whether the bottle contained tylosin tartrate or placebo was indicated in a randomization list kept by an external study manager. One-third of the bottles contained placebo and two-thirds tylosin tartrate. The test products were tylosin tartrate (Tylosin tartrate tablets, University Pharmacy, Helsinki, Finland) tablets 120 mg and 240 mg containing 100 mg and 200 mg of tylosin, respectively, and placebo tablets containing microcrystalline cellulose (Microcrystalline cellulose tablets, University Pharmacy, Helsinki, Finland). The tablets were identical visually, as were the labels on the bottles.

On day 3 of the treatment period, the owner was contacted by telephone to determine the clinical condition of the dog. If the dog had excessive diarrhea and the clinical condition was poor, the dog was released from the study and treated accordingly.

For dogs continuing the protocol, on day 7 of the treatment period, a physical examination, rectal palpation, and evaluation of fecal consistency according to the guidelines were performed and the treatment code broken. To evaluate whether the treatment was effective, the mean value of the fecal consistency scores during the fifth, sixth, and seventh days of treatment was calculated. A responder was defined as having a mean fecal consistency score ≤3.0, and a nonresponder as having a mean fecal consistency score >3.0. The owners were unaware of these definitions. In dogs that did not respond to 25 mg/kg tylosin once a day, the dose was doubled (25 mg/kg tylosin twice a day for seven days). Those not responding to the placebo treatment were given 25 mg/kg tylosin once a day for seven days. Placebo responders were released from the study (Figure 1).

### Statistical methods

To test for differences between the tylosin and the placebo group in the proportion of responders, Pearson's Chi-squared test and Fisher's exact test were applied. The level of significance was set at p < 0.05. Statistical analyses were performed using SPSS 14.0 for Windows.

## Results

Seventy-one suspected TRD dogs (41 males, 30 females) were enrolled in the study. The dogs consisted of 33 different breeds, the most common breeds being Rough Collie (n = 10), German Shepherd Dog (n = 7), and Golden Retriever (n = 6). Dogs' ages at the time of the initial examination ranged from six months to 13 years (median three years and four months). The weight of the dogs ranged from 6.2 kg to 67.2 kg (median 25.5 kg).

**Figure 2:**
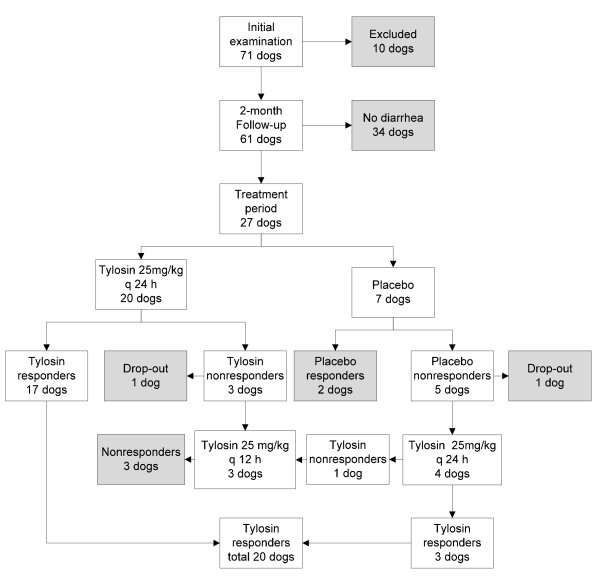
**Results of enrollment, allocation, and response to treatment of suspected TRD dogs**.

At the initial examination, all dogs were randomly allocated into a tylosin treatment group (n = 47) or a placebo group (n = 24). However, ten dogs did not fulfill the selection criteria and were excluded from the study. Sixty-one dogs met the selection criteria and started the two-month follow-up. In 34 dogs, diarrhea did not recur during the follow-up period, and they were released from the study (Figure 2). In 27/61 dogs (43.3%), diarrhea recurred, on a median of day 8 (range 1-60) of the follow-up, and they started the prospective treatment period. At the time of commencement of the treatment period, the tylosin group comprised 20 dogs and the placebo group 7 dogs.

The 27 dogs starting the treatment period consisted of 19 different breeds, the most common breeds being Golden Retriever (n = 5), German Shepherd Dog (n = 3), and Rough Collie (n = 3). Both the tylosin and placebo groups contained more males than females. The age of the dogs ranged from six months to 11 years (median three years). The weight of the dogs ranged from 8.2 kg to 64.0 kg (median 24.5 kg).

In the 27 dogs starting the treatment period, the clinical signs during earlier diarrhea episodes were similar in dogs of both groups (Table 1). Diarrhea was mainly watery or pulpy, and blood or mucus was often present. The frequency of defecation was increased in 81% of the dogs and weight loss was present in an equal proportion. Vomiting occurred in 11/27 dogs. In more than half of the dogs, diarrhea signs started at a young age (<2 years). In 63% of the dogs, tylosin treatment had been discontinued at least twice, and diarrhea had recurred in 55% within 20 days of discontinuation. In all dogs, diarrhea had ceased within two days of the start of tylosin treatment.

**Table 1:** Clinical history prior to the study of the 27 dogs starting the treatment period.

	Tylosin group		Placebo group	
	
	Responder (n = 17)	Nonresponder (n = 3)	Responder (n = 2)	Nonresponder (n = 5)
**Diet at initial examination**				
Commercial	2	2		2
Home-made	6	1	1	1
Both	9		1	2
**Commercial diet**				
Dry	8	1		2
Canned	1			
Both	2	1	1	2
**Signs during diarrhea episodes**:				
**Appetite**				
Normal	11			
Moderately decreased	1	2	1	2
Severely decreased	3	1		3
Increased	2		1	
**Weight loss**				
Moderate	5		1	3
Mild	8	2	1	2
None	3	1		
No answer	1			
**Vomiting**				
Typical	2	2		2
Fairly typical	3		1	1
None	11	1	1	2
No answer	1			
**Borborygmus**				
Typical	5	2	1	3
Fairly typical	6	1	1	1
None	6			1
**Flatulence**				
Typical	9	1	1	3
Fairly typical	1	1		
None	7	1	1	2
**Fecal consistency**				
Watery	15	3	1	4
Bloody	7			4
Mucus	9	1	1	5
Pulpy	9	2	2	2
**Frequency of defecation**				
Increased	14	2	2	4
Normal	2	1		1
No answer	1			
**Age in years when diarrhea began**				
< 1	6	2	1	4
1-2	3		1	
3-7	7	1		1
> 7	1			
**Tylosin discontinued n times prior to study**				
0	1	2		
1	3	1	1	2
2-4	10		1	
> 4	3			3
**Cessation of diarrhea after start of tylosin treatment**				
1 day	10	2		5
2 days	7	1	2	
**Recurrence of diarrhea after tylosin discontinuation**				
< 10 days	5	2	1	1
10-20 days	4			2
> 20 days	2			1
Irregular	4		1	1
No recurrence	2	1		

In dogs starting the treatment period, CBC and serum biochemical profile were within the reference range or there were only minor changes at the initial examination. Serum cobalamin concentrations were below the reference range in eight dogs, six in the tylosin and two in the placebo group. Serum folate concentrations were below the reference range in six dogs, five in the tylosin and one in the placebo group. Seven dogs, all in the tylosin group, had serum folate concentrations above the reference range.

There were no abnormal findings in urine samples.

Fecal samples of six dogs, four in the tylosin group, all being responders, and two in the placebo group, one being a responder and one a nonresponder, were positive for *Giardia *spp. One dog had hookworms and one had isospora, both in the tylosin group.

Gastroduodenoscopy was performed on 19/20 dogs of the tylosin group and 5/7 dogs of the placebo group. In three dogs, gastroduodenoscopy was not performed at the owner's request. In one dog, the duodenum was not reached. Table 2 displays the histopathological scoring of duodenal changes in endoscopically taken mucosal biopsies in dogs receiving either tylosin or placebo. The category "other types of inflammation" included three dogs with mild to moderate neutrophilic inflammation and one dog with lymphohistiocytic inflammation. Mild morphological changes were found in 12 biopsies, all of which showed mild or moderate inflammation and were therefore considered part of the inflammatory reaction. Due to the small number of dogs in the placebo group, no statistical analysis was possible between the groups concerning the results of the histopathological examination of the biopsies.

**Table 2:** Histopathological scoring of biopsies according to responders and nonresponders in the tylosin and placebo groups.

	Tylosin group		Placebo group	
	Responder (n = 16)	Nonresponder (n = 3)	Responder (n = 2)	Nonresponder (n = 2)
**Type of inflammation**				
No changes	3	0	0	1
Lymphoplasmacytic	9	2	1	0
Eosinophilic	2	1	0	0
Other	2	0	1	1
				
**Grade of inflammation**				
No changes	3	0	0	1
Mild	9	1	1	1
Moderate	4	1	1	0
Severe	0	1	0	0

When diarrhea appeared in the 27 dogs and the treatment period started, a physical examination was performed and blood and fecal samples were taken. None of the dogs was in poor clinical condition, but five dogs had decreased appetite. Four dogs were slightly apathetic at the beginning of the diarrhea. Flatulence was present in 14 dogs. Eleven dogs suffered from borborygmus and five from vomiting. The results from the blood samples did not differ from those at the initial examination. *Giardia *spp. or other endoparasites were not detected in any of the fecal samples.

In the bacteriological analysis, fecal samples were positive in 18/27 dogs for *Clostridium perfringens *(nine tylosin responders, three tylosin nonresponders, two placebo responders, four placebo nonresponders), in 2/27 dogs for *Campylobacter jejuni *(one placebo responder, one nonresponder), in 1/27 dogs for *Yersinia enterocolitica *(placebo nonresponder), and in 1/27 dogs for *Salmonella Typhimurium *(tylosin responder). In two dogs, two pathogenic bacteria were detected simultaneously. No difference was seen in the bacteriological culture of *Clostridium perfringens *between the responders and nonresponders (Pearson's Chi-squared test p = 0.136, Fisher's exact test two-sided p = 0.201) or between the tylosin and placebo group (Pearson's Chi-squared test p = 0.214, Fisher's exact test two-sided p = 0.363).

In the tylosin group, 17/20 dogs responded to treatment (Figure 2), with a mean fecal score over the last three treatment days of 2.47 (range 1.83-2.93). Three dogs did not respond to the tylosin therapy, with a mean fecal score over the last three treatment days of 3.64 (range 3.41-3.86). After the treatment period, one of these three dogs was removed due to protocol violation (Figure 2).

In the placebo group, 2/7 dogs responded to placebo treatment (Figure 2), with a mean fecal score over the last three treatment days of 2.68 (range 2.67-2.70). These dogs were released from the study. Five dogs did not respond to the placebo treatment, with a mean fecal score over the last three treatment days of 3.89 (range 3.19-5.00). One of them was removed after the treatment period for noncompliance. The four remaining nonresponders were subsequently given tylosin 25 mg/kg once daily. Three of these dogs responded; the mean fecal score over the last three treatment days was 2.36 (range 2.14-2.50). One dog did not respond, with a mean fecal score over the last three treatment days of 3.58 (Figure 2).

Two nonresponders in the tylosin group and one in the placebo group that failed to respond to tylosin 25 mg/kg once daily subsequently received tylosin 25 mg/kg twice daily (Figure 2). None responded to the double dose, the mean fecal score over the last three treatment days being 3.48 (3.29-3.67).

The proportion of dogs with a fecal consistency score of three or less was 85% (17/20) in the tylosin group and 29% (2/7) in the placebo group. This difference is significant (Pearson's Chi-squared test p = 0.0049 and Fisher's exact test two-sided, p = 0.0114).

Overall, 20/24 dogs (83%) responded to tylosin therapy and 4/24 (17%) did not (Figure 2).

## Discussion

Tylosin has been widely used to treat canine intermittent and chronic diarrhea, although only a few studies have been published about the effect of tylosin on dogs with diarrhea [1,2,7,8]. None of the studies cited was controlled with a placebo. To provide more evidence-based research on tylosin, we performed a placebo-controlled, randomized, double-blinded, prospective clinical trial. Of the 24 suspected TRD dogs treated with tylosin, 20 were responders. The outcome of these dogs was in agreement with owners' earlier experiences. However, in four dogs diarrhea did not stop with tylosin medication, although based on information from the owners it had previously helped to control diarrhea. At this point, when the etiology of TRD is unclear, it is possible that the reasons for the diarrhea in these dogs differed from the previous times.

Our trial showed that the effectiveness of tylosin on fecal consistency of dogs with suspected TRD is significantly higher than that of placebo treatment. In 85% of dogs receiving tylosin, diarrhea ceased within a seven-day treatment period, in contrast to a response rate of 29% in dogs receiving placebo. Interestingly, the effect of tylosin on these dogs is similar to that reported in a study with rhesus macaques suffering from chronic diarrhea and failing to respond to antibiotics other than tylosin [9].

In healthcare, efficacy is usually defined as the capacity for beneficial change of a given intervention and not only as a curative effect. Based on our results, tylosin is effective in treating chronic diarrhea in dogs. However, as the etiology of TRD remains obscure, we do not know whether tylosin is a symptomatic or curative treatment. After discontinuation of tylosin treatment, diarrhea recurs in many dogs, and thus, tylosin could be considered a symptomatic treatment in these dogs. However, our findings are in accordance with previous studies [1,2] and anecdotal reports in that not all dogs develop diarrhea after stopping the tylosin course; in these patients, tylosin could be seen as a curative treatment.

To our knowledge, no other double-blinded, placebo-controlled studies on dogs with diarrhea exist in the literature. Performing this kind of a trial with client-owned diarrhea patients is demanding. The most difficult part is ensuring that at the start of the trial both placebo and active treatment groups are as homogeneous as possible. Unfortunately, dogs suffering from diarrhea usually comprise a very heterogeneous group of patients. Assuring the similarity of both groups concerning clinical history, clinical signs, and physical examination is therefore challenging. Using only dogs with suspected TRD, based on previous outcomes of therapeutic trials with tylosin, provided a similar starting point for all dogs at the beginning of the trial. The suspected TRD dogs participating in this study represented dogs that had received at least one empiric tylosin treatment due to recurrent diarrhea during the last four weeks prior to study enrollment and the owners had the impression that tylosin had had a positive effect on their dogs' diarrhea. Furthermore, the absence of diarrhea at the beginning of the trial was set as one selection criterion. Based on the initial examination, we could not predict whether the diarrhea would reappear in follow-up or whether the dog would respond to tylosin within the treatment period. Using dogs that have previously received tylosin for the treatment of their diarrhea is a reliable regime to explore whether the anecdotal reports of owners' earlier positive impressions of the tylosin therapy in treating their dogs' diarrhea are true.

Difficulties in this kind of trial are encountered also in keeping owners committed to the study protocol while their pet suffers from diarrhea longer than necessary. Skewed randomization strategies, which allow more patients to enter one group versus another, are a recognized approach when there is an ethical risk of severe clinical signs in patients not receiving the active treatment [10-12]. The allocation ratio of 2:1 minimized inconvenience and discomfort of patients not receiving the active treatment and having severe clinical signs. Excessive diarrhea can result in a patient's poor clinical condition due to severe dehydration and electrolyte and acid-base imbalances. Fortunately, none of the dogs enrolled in our study had such excessive diarrhea that the trial had to be interrupted because of inconvenience to the owner. In no case did the clinical condition of patients worsen to the extent that use of a humane end-point and release from the trial were essential.

The dogs were randomized into the placebo and tylosin groups already at the initial examination, when the dogs had no clinical signs of diarrhea. This was done because we assumed, based on a previous study on TRD [1], that during the two-month follow-up diarrhea would reappear in almost every dog enrolled in the study and therefore in similar numbers of dogs in both groups. However, diarrhea reappeared in only every second dog enrolled in the study and, by chance, less frequently in the dogs assigned to the placebo group. For this reason, the anticipated distribution to the different treatment groups did not come to fruition, and at the time of the treatment the placebo group comprised fewer dogs than expected. In retrospect, randomization to the placebo and tylosin groups should have taken place at the time-point when diarrhea started and the treatment period began.

Concerning the lower recurrence rate of diarrhea in the dogs enrolled in our study, it is pertinent that we included also dogs that had received only one successful tylosin course due to recurrent diarrhea of unknown origin, in contrast to previous studies [1,2], which consisted of dogs receiving several tylosin courses and had a repeated response. Our study also differed from these earlier studies in that we investigated a far higher number of dogs, which can lead to speculation that we may have included patients with either a broader variety of etiologies or differing severities of the same disorder, resulting in different responses to tylosin. It remains unclear why certain dogs do not develop diarrhea again after discontinuing tylosin.

The length of the treatment period was set at seven days because previous studies have shown that with tylosin treatment diarrhea stops within a few days [1,2]. No official dose recommendations exist for tylosin in the treatment of canine enteropathies. The dose of 25 mg/kg once daily used in the treatment period here is similar to that suggested in the literature [13]. Based on our results, this dose appears sufficient, and no additional benefit was gained by increasing it. None of the nonresponders subsequently receiving tylosin 25 mg/kg twice daily responded to the double dose.

The clinical history of suspected TRD dogs was similar to that reported in a smaller study consisting of 14 dogs [1]. The dogs in our study were young or middle-aged and belonged mainly to medium-sized and giant breeds, but no breed predilection was found. The clinical signs suggested the diarrhea was more often large bowel diarrhea, as in more than two-thirds of the dogs the frequency of defecation had increased and about half of the dogs had mucus and bloody feces. The histopathologic changes in the mucosal biopsies varied between the different dogs, but due to the small number of dogs in the placebo group, no statistical analysis was possible between the groups.

No changes specific to TRD were detected in the histopathological examinations, and therefore, one could not based on the histopathology predict whether the dog would respond to tylosin treatment or not. At the initial examination, no reason for the diarrhea in the dogs was found. The dogs could thus be referred to as dogs with diarrhea of unknown origin.

Fecal samples were cultured when the diarrhea started, and 20/27 samples were positive for some of the most common enteropathogenic bacteria. The positive findings were distributed across responders and nonresponders in both treatment groups, indicating that these pathogenic bacteria are unlikely to be the causative factors for the diarrhea in TRD dogs. Enteropathogenic bacteria have been detected also in the fecal cultures of clinically healthy dogs [3] and dogs with TRD [1,2]. Fecal samples of six dogs were Giardia-positive at the time of the initial examination. All of these dogs were negative for Giardia after fenbendazole medication when the control sample was taken during diarrhea recurrence in the follow-up period. The highest sensitivity and specificity for *Giardia *spp. were detected for the immunoassay we utilized in our trial, when three different methods were compared [14]. We can therefore conclude that *Giardia *spp. is not a possible etiologic agent for the diarrhea in these dogs.

Different antibiotics have been recommended for the treatment of canine chronic and intermittent diarrhea [15]. However, no study has been carried out to determine which antibiotic is the most effective in treating chronic diarrhea patients. In one study with six dogs, tylosin was found to be superior to trimethoprim sulfonamide, doxycycline, or metronidazole [2]. No apparent tylosin-associated clinical side-effects have been described. No clinical adverse events occurred during our trial. Interestingly, the effect of tylosin has been reported not to diminish even with longer or repeated treatment periods [13]. Long-term usage of antibiotics typically contributes to the development of microbial resistance to antibiotics, and getting a positive effect when the same antibiotic substance is used repeatedly to treat the same disease in the same individual is difficult. Tylosin, as all antibiotics, causes resistance in the intestinal bacterial flora. This is a significant problem and chronic diarrhea in dogs should preferably be treated without long-term antibiotic administration. Indiscriminate use of antibiotics should be avoided, and in patients with chronic diarrhea every effort should be made to achieve a diagnosis that enables a specific therapy. An extensive work-up to rule out systemic disorders with secondary diarrhea, endoparasites, and food-responsive diarrhea is recommended before starting a therapeutic trial with tylosin.

## Conclusions

Our findings indicate that tylosin is effective in treating recurrent diarrhea in dogs. The dose of 25 mg/kg once daily seems sufficient. No changes specific to TRD were detected in the examinations. Several different underlying reasons may exist for TRD. Currently, it is difficult to predict which patients suffering from gastrointestinal disease will benefit from tylosin medication. The only way of knowing appears to be a therapeutic trial. The exact mechanism of action of tylosin is unknown and further studies are needed to explore why tylosin has a favorable effect in the treatment of canine chronic enteropathies.

## Competing interests

The authors declare that they have no competing interests.

## Authors' contributions

SK was the principal researcher of the study, made the practical arrangements, carried out the clinical examinations and together with TSp the gastroduodenoscopies, and drafted the manuscript. TSp made a substantial contribution to the design of the study, carried out together with SK the gastroduodenoscopies, and helped draft the manuscript. PS carried out the histopathological examinations of the mucosal biopsies of the gastrointestinal tract. TSk conducted the bacteriological analysis of the fecal samples. ML helped perform the clinical examinations together with SK. EW made a substantial contribution in the design of the study and assisted significantly in drafting the manuscript. All authors read and approved the final manuscript.
